# A study of segment weight optimization with the CMS XiO step‐and‐shoot IMRT technique for prostate cancer

**DOI:** 10.1120/jacmp.v13i1.3622

**Published:** 2012-01-05

**Authors:** Ramachandran Prabhakar, Jim Cramb, Christopher Gehrke, Justin Anderson, Judy Andrews

**Affiliations:** ^1^ Physical Sciences Peter MacCallum Cancer Centre Victoria Australia; ^2^ Radiotherapy Services Peter MacCallum Cancer Centre Victoria Australia

**Keywords:** treatment planning, IMRT, XiO, segmented weighted optimization, prostate cancer

## Abstract

The aim of this study was to compare IMRT optimization in the CMS XiO radiotherapy treatment planning system, with and without segment weight optimization. Twenty‐one prostate cancer patients were selected for this study. All patients were initially planned with step‐and‐shoot IMRT (S‐IMRT). A new plan was then created for each patient by applying the segment weight optimization tool (SWO‐IMRT). Analysis was performed on the (SWO‐IMRT) and (S‐IMRT) plans by comparing the total number of segments, monitor units, rectal and bladder dose. The study showed a statistically significant reduction in the total number of segments (mean: 25.3%; range: 16.8%–31.1%) with SWO‐IMRT as compared to S‐IMRT (p<0.0001). Similarly, a mean reduction of 3.8% (range: 0.4%–7.7%) in the total MU was observed with SWO‐IMRT (p<0.0001). The study showed an average rectal dose decrease of 13.7% (range: 7.9%–21.4%) with SWO‐IMRT (p<0.0001). We also observed a statistically significant reduction of 26.7% (range: 16.0%–41.4%; *p* < 0.0001) in the mean dose to the posterior one‐third rectum and an overall reduction in mean bladder dose of 2.2% (range: 0.1%–6.1%) for SWO‐IMRT (p<0.0001). This study shows that the segment weight optimization method significantly reduces the total number of segments and the dose to the rectum for IMRT prostate cancer. It also resulted in fewer monitor units for most of the prostate cases observed in this study.

PACS numbers: 85.55.ne; 87.55.de; 87.55.kd

## I. INTRODUCTION

Prostate cancer is the most common cancer in Australian men and is the second most common cause of cancer deaths in men after lung cancer. One in nine men in Australia develop prostate cancer in their lifetime, and one in five have a risk of developing prostate cancer by the age of 85. Around 20,000 new cases are diagnosed in Australia each year, of which close to 3300 men die of prostate cancer, which is equivalent to the annual number of women who die from breast cancer.^(^ ^1^ ^)^ Radiotherapy plays an important role in widening the gap between the number of men diagnosed with prostate cancer and the number of men who die from the disease. Furthermore, advanced radiotherapy treatment techniques such as intensity‐modulated radiation therapy (IMRT) and image‐guided radiation therapy (IGRT) help to reduce the radiotherapeutic toxicity.^(^ ^2^ ^)^ IMRT has been shown to reduce normal tissue toxicity as compared to 3D CRT.^(^ ^3^ ^–^ ^5^ ^)^ However, the most important limiting factors in dose escalation of prostate cancer radiotherapy are bladder and rectal toxicities. Pollack et al.^(^ ^6^ ^)^ have shown that an increase in the prescribed dose from 70 Gy to 78 Gy results in a highly significant improvement in freedom from failure for intermediate to high‐risk prostate cancer patients, but with an increase in rectal toxicity. Long‐term results of a dose escalation trial have also shown that the clinical failure rate was significantly reduced with dose escalation, and the complication rate could be considerably decreased by reducing the amount of treated rectum.^(^ ^7^ ^)^

The step‐and‐shoot technique is one method of delivering IMRT whereby each field is delivered as a sequence of static MLC field segments. The shapes and the monitor unit weights of the segments are designed by the MLC segmentation algorithm. The algorithm converts the smooth intensity maps of the optimal fluence to a deliverable fluence; however, the conversion process degrades the quality of the final fluence. Higher monitor units (MU) and segments in an IMRT plan mean increased treatment times and greater equipment wear and tear. Higher MU may also increase the probability of secondary malignancies after radiotherapy treatment. A study by Que^(^ ^8^ ^)^ shows that scatter radiation, cumulative leakage, and non‐deliverable fractional MU can be reduced by decreasing the number of MLC segments per treatment. Several algorithms have been proposed to reduce the number of segments.^(^ ^9^ ^–^ ^11^ ^)^ The segment weight optimization (SWO) engine was introduced by CMS with the release of XiO version 4.50.00. The SWO tries to increase the similarity between the segmented and the optimized plan and decreases the total number of segments.

This study has been framed to assess the use and quantify the advantage of segment weight optimization for IMRT planning of prostate radiotherapy, using the CMS XiO treatment planning system. The study compares the IMRT optimization with and without segment weight optimization.

## II. MATERIALS AND METHODS

**Figure 1:**
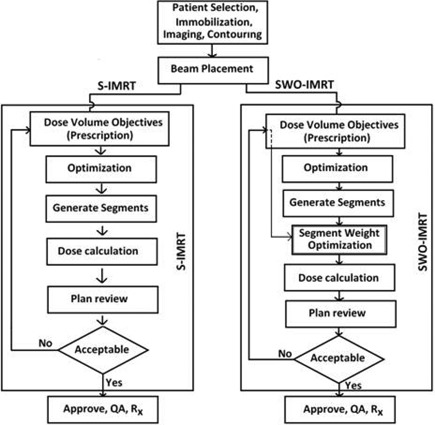
Flow chart of S‐IMRT and SWO‐IMRT optimization methods.

Twenty‐one prostate cancer patients were selected for this study. All patients underwent a planning CT scan using a Philips Brilliance Big Bore CT (Philips Healthcare, Andover, MA). Patient preparation prior to CT included the consumption of 750 ml of fluid 30 minutes prior to the scan which was repeated on every treatment day. Planning was performed using the CMS XiO treatment planning system (Version 4.51.02) (Elekta, Stockholm, Sweden). A seven‐field IMRT plan with beams angles of 25°, 75°, 125°,180°, 235°, 285°, and 335° (IEC convention) was generated for each patient. A PTV dose of 78 Gy in 39 fractions was prescribed. Figure 1 shows the steps involved in S‐IMRT and SWO‐IMRT. Both methods use the same dose constraints and follow identical steps at the start of the process. However, SWO‐IMRT includes the addition of segment weight optimization which is performed immediately following the generation of the segments. The IMRT optimization parameters are given in Table 1. The IMRT guidelines for the treatment plans were: 99% of the PTV should be covered by 95% of the prescribed dose; mean dose to the PTV should be between 77.5 Gy and 78.5 Gy; whenever the PTV and rectum overlap, the 74.1 Gy (95%) isodose line should cover the PTV posteriorly, and the 77.2 Gy (99%) isodose line should be anterior to the anterior edge of the rectum; the maximum dose to the bladder should be less than 70 Gy; the 37 Gy isodose line should be above the posterior one‐third rectum on most slices; the global dose maximum should lie within the volume of PTV that excludes rectum and should not be larger than approximately 82 Gy.

**Table 1:** IMRT optimization parameters.

	*Objective*	*Dose (Gy)*	*Volume (%)*	*Weight*
Overlap (Target)	Maximum	74.1	0	400
	Minimum	70.3	100	600
Post‐rectum	Maximum	37	0	200
CTV	Maximum	79	0	200
	Minimum	78	100	500
PTV	Maximum	78	0	300
	Minimum	76.4	100	700
Bladder	Maximum	70	0	200
External	Maximum	39	0	200
Rectum	Dose Volume	63	23	100
	Dose Volume	53	30	100
	Dose Volume	35	45	200
	Dose Volume	25	60	100
	Dose Volume	17.5	70	200

The XiO optimizer minimizes the cost function using a conjugate gradient optimization algorithm (a special type of gradient descent optimization algorithm) to search for the minimum value of the cost function.^(^ ^12^ ^,^ ^13^ ^)^ The optimization process consists of a series of iterations where the optimizer updates the beamlet doses until the current dose distribution is significantly closer to the dose objectives than the previous iteration. XiO defines the objective function as:

(1) $$O=w^{*}{\left(D_{current}-D_{prescribed}\right)}^{p}$$

where *w* is the weight and *p* is the power, and $D_{\mathrm{current}}$ and $D_{\mathrm{prescribed}}$ are the current and prescribed doses to a voxel, respectively. The sum of the prescribed objective functions for all the targets and organs at risk (OAR) comprises the score function and is a measure of how close the dose distribution is to the user defined dose objectives. The weight determines the relative importance of objectives — increasing the weight increases the importance of a structure/organ dose constraint. After optimizing and reviewing the results, the weight can be adjusted to help achieve the target objectives. The power is similar to the weight, but a change to the power has a greater effect. Typical values are between 2.0 and 2.7. It emphasizes the hard constraints on the maximum or minimum objectives, and increases the penalty on voxels that have doses in violation to a structure's dose volume objective. The hotspots within the target and OAR can be reduced by increasing the power. It is possible that large power values can cause unusual beam weightings or beams to be turned off altogether. In such situations, a value of zero for the beam weight maximum iterations can be entered to disable the beam weight optimization.

XiO also accounts for the scatter during the optimization. The length of the scatter tails is controllable with a parameter called scatter extent, which is the distance any beamlet contributes dose beyond its geometric edges. If the scatter extent is zero, the optimization algorithm completely ignores the scatter. More information is tracked with larger scatter extent (tails), resulting in a better optimized plan at the cost of increased optimization time. The optimization of beamlets is restricted to an area around the target volume by applying an optimization margin. The optimization margin accounts for the penumbra and ensures the beamlets intersect up to and slightly beyond the target. This is important because any beamlets falling beyond the projection of this margin, in a beam's eye view, will not be included in the optimization. A larger margin may result in higher doses to the surrounding critical structures, and a smaller margin may result in decreased dose (cold spots) at the edges of the target volume. Hence, the choice of margin is important. Whenever structures overlap, the optimization engine uses a ranking system to determine which structure owns each voxel. For example, if the PTV has a rank of 1 and the rectum has a rank of 2, then priority is given to the PTV constraints. Voxels can be shared if ranks are not exclusive. This allows several structures to have the same ranking. If the structures don't overlap, rank does not affect the optimization.

The goal of the optimization process is to find the best combination of beamlet intensities or weights that produces a minimum cost function. Each iteration requires a gradient calculation that takes the derivative of the cost function with respect to the intensity of each beamlet. This is followed by a line search for a minimum cost function in the direction of the negative gradient. The cost function should decrease with successive iterations. The optimization process ends after the user‐defined number of iterations is exceeded, or when the cost function converges to the solution. XiO uses a convergence criterion that sets the termination point for the IMRT optimization iteration. Termination occurs when the difference in the score function from one iteration to the next falls below the convergence criterion. A lower convergence criterion requires higher number of iterations to be performed by the optimization engine. XiO performs segmentation of the fluence map into an MLC sequence during the final stage of the optimization. If the optimization constraints or sequencer settings are changed during the optimization process, XiO resets the fluence map and restarts from the beginning of the optimization process. After the initial optimization and segmentation process, XiO computes the plan and optimizes the beam weights using the beam weight optimization. The beam weight optimization employs the intensity maps that have been generated during the optimization process and reweights all the beams based on the same prescription used for the initial optimization. It optimizes only the beam weights and not the individual segment weights during this process of optimizing the loss between the optimized plan and segmented plan.

Table 2 shows the optimization control parameters for S‐IMRT and SWO‐IMRT used in this study. In the optimization engine for S‐IMRT, a step increment of 0.5 cm was used along the x‐axis which controls the beamlet width at the isocenter. A 1.0 cm scatter extent and a 0.5 cm optimization margin were also defined in the optimization settings. For the initial optimization and for the beam weight optimization, the maximum number of iterations was set to 60, with a convergence criterion of 0.0001%. In S‐IMRT, a sliding window technique was used to generate the segments, with 10 discrete intensity levels and with a minimum segment size of 1.0 cm. The final dose calculation was performed using the multigrid superposition (MGS) algorithm with 2 mm voxel spacing. IMRT plans (S‐IMRT) were generated for all 21 patients.

**Table 2:** Optimization control parameters for S‐IMRT and SWO‐IMRT.

	*S‐IMRT*	
Step increment (cm)		0.5
Iteration between DVH update		10
	*Initial Optimization*	
Convergence criterion (%)		0.0001
Maximum iterations		60
Scatter extent (cm)		1
Optimization margin (cm)		0.5
	*Beam Weight Optimization*	
Convergence criterion (%)		0.0001
Maximum iterations		60
	*SWO‐IMRT*	
SWO grid spacing (cm)		0.3
Convergence criterion (%)		0.0001
Maximum iterations		100
Reverse iterations		5

**Figure 2:**
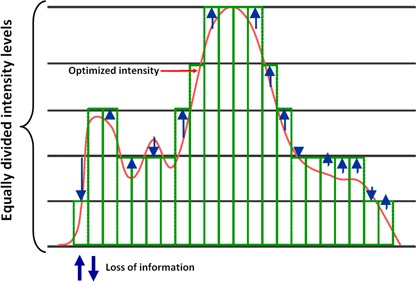
Loss of information with equally divided intensity levels.

A new plan (SWO‐IMRT) was created from S‐IMRT using the segment weight optimization method. Unlike the beam weight optimization in S‐IMRT, the SWO method optimizes the individual segment weights. With S‐IMRT, the optimized intensity levels are divided into equal intensity levels. Figure 2 shows the optimized intensity and the loss of information with equally divided intensity levels.^(^ ^14^ ^)^ It clearly illustrates that by generating unequal intensity levels it is possible to achieve closer to the optimized intensity levels. The segment weight optimization (SWO) performs this by reweighting the segments resulting in unequal intensity levels. It also eliminates the MU for individual segments that fall below a set minimum MU per segment and re‐optimizes the remaining segments. This reduces the total number of segments, shortening the treatment time. The user‐controlled parameters for SWO optimization are: SWO grid spacing, convergence criterion, maximum iterations, reverse iterations, and minimum segment MU. SWO passes through a major cycle and several revision cycles. The major cycle performs dose calculation for all segments. In the SWO optimization engine, a maximum of 100 iterations was used with five revision iterations in this study (Table 2). The major part of the optimization process occurs in the first 30 iterations and after this, relatively small improvements are made to the plan. A 3 mm grid spacing was set in the optimization engine. The minimum MU segment was restricted to 5 MU and the convergence criterion was set to 0.0001%. SWO reads the segment dose files and performs the major cycle (iterations) before performing the revision iterations. XiO calculates the segment weight per segment for each beam and indicates the segments that are left to calculate. The segment weights are then optimized based on the IMRT prescription values. The fast version of the multigrid superposition (fMGS) algorithm was used to speed up the SWO calculation. After the dose computation, each plan was analyzed, utilizing XiO's dose‐volume histogram (DVH) tools. If the target dose or any organ at risk (OAR) dose constraint was not met, the IMRT prescription was changed, and segment weight optimization was performed as shown in Fig. 1. The final dose calculation was performed with 2 mm grid spacing. Analysis was performed with (SWO‐IMRT) and without (S‐IMRT) segment weight optimization method. The plans were compared for the total number of segments, monitor units, and doses to rectum, posterior one‐third rectum and bladder. A paired sample t‐test to compare the means of the above dosimetric parameters was performed, and the statistical significance was determined by p‐value (p<0.05).

## III. RESULTS

**Figure 3:**
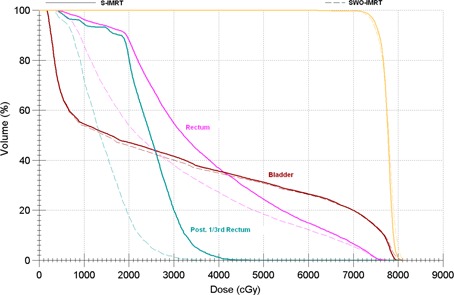
Comparision of dose‐volume histogram of a prostate IMRT plan, using S‐IMRT and SWO‐IMRT.

**Figure 4:**
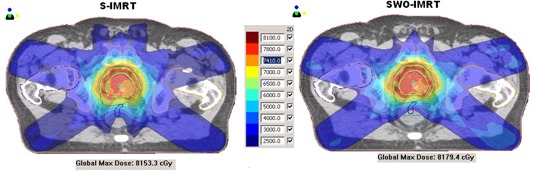
Comparison of dose distribution for S‐IMRT and SWO‐IMRT.

Figure 3 compares a dose‐volume histogram (DVH) of S‐IMRT and SWO‐IMRT for a randomly selected patient, and Fig. 4 shows the isodose colorwash for the same patient. It is evident from the DVH and from the Fig. 3 that dose to the posterior rectum is significantly reduced with SWO‐IMRT. Table 3 shows the comparison of dosimetric parameters between S‐IMRT and SWO‐IMRT plans. There is a statistically significant reduction in the total number of segments (mean: 25.3±3.9%; range: 16.8%–31.1%) with SWO‐IMRT as compared to S‐IMRT (p<0.0001). Similarly, a mean reduction of 3.8±2.1% (range: 0.4%–7.8%) in the total MU was observed with SWO‐IMRT (p<0.0001). The study showed a mean rectal dose decrease by 13.7±3.1% (range: 7.9%–21.4%) with SWO‐IMRT (p<0.0001). There is also a statistically significant reduction of 26.7% (range: 16.0%–41.4%) in the mean dose to the posterior one‐third rectum for SWO‐IMRT as compared to the S‐IMRT (p<0.0001). The overall reduction in the mean bladder dose was 2.2±1.7% (range: 0.1%–6.1%) for SWO‐IMRT.

**Table 3:** Comparison of dosimetric parameters between simple optimization and segment weighted optimization.

	*S‐IMRT*		*SWO‐IMRT*	
*Parameters*	Mean±Stdev.	*Range* (min−max)	Mean±Stdev.	*Range* (min−max)
No. of Segments	119.5±12.8	101–151	89.1±9	73–107
No. of Monitor Units	786.4±93.1	653–989	755.8±87.3	623–953
Mean Dose to Bladder (cGy)	2909.4±1007.1	1332–5105	2839.5±954.4	1323–4886
Mean Dose to Rectum (cGy)	3697.5±259.5	3298–4225	3192.2±262.2	2737–3773
Mean Dose to Post‐one–third Rectum (cGy)	2438.1±396.4	1684–3566	1792.3±374.6	1260–2997

## IV. DISCUSSION

Dose escalation has been proven to improve tumor control probability, especially in the treatment of prostate cancer. The main limiting factor for dose escalation is dose to the critical structures. Every effort should be made to keep the dose to the rectum within its tolerance limits in order to minimize the risk of long‐term complications from radiotherapy. These include rectal bleeding, fistula, fecal incontinence and rectal discomfort. Several methods have been proposed to reduce the dose to the rectum including use of a rectal balloon,^(^ ^15^ ^)^ spacer,^(^ ^16^ ^)^ and human collagen^(^ ^17^ ^)^ inserted between the rectum and prostate. Sushil et al.^(^ ^16^ ^)^ have shown that increasing the physical separation of the rectum from the prostate by injecting a spacer reduces the rectal radiation dose. Recently, Noyes et al.^(^ ^17^ ^)^ have shown that human collagen injections reduce the dose to the anterior rectum by 50%. Treatment position was also found to affect dose to the rectum.^(^ ^18^ ^)^ Another important strategy to reduce the rectal dose is the use of image‐guided radiotherapy^(^ ^19^ ^,^ ^20^ ^)^ to enable a reduction of the CTV to PTV margin.

Intensity‐modulated radiotherapy plays a vital role in the treatment of prostate cancer by reducing rectal toxicity and thereby providing an opportunity for dose escalation. In step‐and‐shoot IMRT, the intensity levels and the number of segments generated are interlinked. More intensity levels require more segments which yield a better resolution. However, more segments can lead to increased treatment time and increased overhead to MLCs. Conversely, fewer intensity levels degrade the final dose distribution. Hence, an optimal intensity level should be used. With S‐IMRT optimization, more segments are generated which prolongs the treatment time required for implementing the treatment plan. The segmentation algorithm also generates small segments and segments with few monitor units. Moreover, individual segment weights are relatively uniform and do not show much variation and may result in segmented plans that differ from optimized plans. The segment weight optimization method generates more nonuniform segment weights, which results in segmented plans that closely match the optimized plan. The SWO uses the basic concept of modifying the uniform intensity levels to nonuniform levels by effectively reweighting the segments during the segment weight optimization process.

SWO uses the same optimizer algorithm and dose constraints as used for the S‐IMRT to optimize the weights of individual segments. The main advantage of SWO is that it eliminates segments that have a small number of monitor units, after which the system optimizes again to achieve the defined goals. The user has direct control over the minimum MU per segment. After SWO is performed once, the user can alter the IMRT prescription page and apply a second SWO calculation, bypassing the segmentation stage to improve plan results. This study shows that the use of the segment weight optimization method not only reduces the total number of segments and MU, but also has an impact on the dose to critical structures in prostate cancer. IMRT significantly reduces the dose to the rectum and paves a way for dose escalation. It also reduces the bladder dose marginally for most patients. In this study, a planning objective was to ensure that the 37 Gy isoline lies above the post‐one–third of the rectum. An important observation from this study is the significant reduction to this posterior one‐third of the rectum, in addition to the dose reduction to the rectum.

A typical issue when running SWO‐IMRT for the first time is the small reduction in PTV dose with an increase in the dose maximum. This may be due to the elimination of segments that fall below a set limit, as XiO automatically deletes any segments that fall below the minimum segment MU. The SWO calculation permits choices, such as direct control over the minimum monitor units per segment as well as post segmentation optimization. The dose compromise to the PTV can be eliminated by reapplying the optimization after adjusting the dose volume objectives for the target volume. The SWO calculation gives increased flexibility with regard to the cycle of optimization, segmentation, and evaluation, thereby allowing greater planning efficiency. Reweighting segments improves the agreement between the optimized plan and the final treatment plan. SWO may not be as helpful if the plan already has fewer segments per beam.

The convergence criterion used with SWO is the same as that used for S‐IMRT optimization. The convergence criterion indicates to the optimization algorithm that the cost function has decreased. A low convergence criterion increases the optimization time. Additionally, computation time can be long with SWO calculations as a full dose calculation is performed for each segment. Smaller grid spacing values requires more computational power; therefore, an optimal grid spacing should be chosen. To reduce the computation time, the calculation volume can be resized by applying a somewhat larger SWO grid spacing and a smaller intensity level to generate the MLC segments. Larger grid spacing reduces the time required to perform dose calculation, but the dose calculation outcome will be less accurate. For this reason a 3 mm grid spacing is recommended. In order to increase the accuracy, the final dose calculation should be performed with 2 mm grid spacing. The standard superposition algorithm uses 8 azimuth and 16 zenith rays which results in 128 ray traces per dose point, whereas the fast superposition algorithm limits the number of zenith rays to six, resulting in 48 ray traces per dose point. This makes fMGS algorithm to be approximately 2.5 times faster than the MGS algorithm with a small (1%–2%) decrease in accuracy. The small loss in dose accuracy also leads to 1%–2% change in MU. Hence, the fMGS algorithm may be used to accelerate the process of dose computation for SWO initially and, consequently, the final dose calculation should always be performed with the MGS algorithm.

IMRT has become a standard treatment modality for prostate cancer due to the significant reduction in long‐term rectal complications.^(^ ^3^–^6^ ^,^ ^21^ ^)^ Recent studies have shown that multimodality imaging plays a vital role in accurately delineating the target volume, thereby helping to reduce rectal and bladder complications.^(^ ^22^ ^–^ ^24^ ^)^ The combined approach of SWO with multimodality imaging and image‐guided radiotherapy may further reduce the dose to the rectum. In this work we have only studied the use of SWO for prostate cancer; however it is likely that this technique will be advantageous for other treatment sites as well.

## V. CONCLUSIONS

This study shows that for prostate cancer, XiO's segment weight optimization method significantly reduces the total number of segments and the rectal dose. SWO‐IMRT also reduces the total number of monitor units, accelerates treatment delivery, and lessens equipment wear and tear.
